# Challenges of nurse delivery of psychological interventions for long-term conditions in primary care: a qualitative exploration of the case of chronic fatigue syndrome/myalgic encephalitis

**DOI:** 10.1186/1748-5908-6-132

**Published:** 2011-12-22

**Authors:** Sarah Peters, Alison Wearden, Richard Morriss, Christopher F Dowrick, Karina Lovell, Joanna Brooks, Greg Cahill, Carolyn Chew-Graham

**Affiliations:** 1 School of Psychological Sciences, University of Manchester, Manchester, UK; 2 School of Community Health Sciences, University of Nottingham, Nottingham, UK; 3 School of Population, Community and Behavioural Sciences, University of Liverpool, Liverpool, UK; 4 School of Nursing, Midwifery and Social Work, University of Manchester, Manchester, UK; 5 Centre for Applied Psychological Research, University of Huddersfield, Huddersfield, UK; 6 School of Community Based Medicine, University of Manchester, Manchester, UK

## Abstract

**Background:**

The evidence base for a range of psychosocial and behavioural interventions in managing and supporting patients with long-term conditions (LTCs) is now well-established. With increasing numbers of such patients being managed in primary care, and a shortage of specialists in psychology and behavioural management to deliver interventions, therapeutic interventions are increasingly being delivered by general nurses with limited training in psychological interventions. It is unknown what issues this raises for the nurses or their patients. The purpose of the study was to examine the challenges faced by non-specialist nurses when delivering psychological interventions for an LTC (chronic fatigue syndrome/myalgic encephalomyelitis [CFS/ME]) within a primary care setting.

**Methods:**

A qualitative study nested within a randomised controlled trial [ISRCTN 74156610] explored the experiences and acceptability of two different psychological interventions (pragmatic rehabilitation and supportive listening) from the perspectives of nurses, their supervisors, and patients. Semi structured in-depth interviews were conducted with three nurse therapists, three supervisors, and 46 patients. An iterative approach was used to develop conceptual categories from the dataset.

**Results:**

Analyses identified four sets of challenges that were common to both interventions: (i) being a novice therapist, (ii) engaging patients in the therapeutic model, (iii) dealing with emotions, and (iv) the complexity of primary care. Each challenge had the potential to cause tension between therapist and patient. A number of strategies were developed by participants to manage the tensions.

**Conclusions:**

Tensions existed for nurses when attempting to deliver psychological interventions for patients with CFS/ME in this primary care trial. Such tensions should be addressed before implementing psychological interventions within routine clinical practice. Similar tensions may be found for other LTCs. Our findings have implications for developing therapeutic alliances and highlight the need for regular supervision.

## Background

The management of long-term conditions (LTCs) has changed considerably over the past decade, resulting in such health problems being principally managed within primary care by general practitioners (GPs) and practice nurses (PNs), with support by specialist services when necessary and available [1]. Rewarding more effective and efficient ways of managing LTCs is a central feature of the current United Kingdom (UK) General Practice contract [2].

A growing evidence base now exists for the effectiveness of a range of psychological interventions that are increasingly important in the management of LTCs including chronic obstructive pulmonary disease [3], rheumatoid arthritis pain [4], and chronic pain [5,6], though this evidence base largely comes from secondary care [7]. Introducing psychological interventions developed and established in secondary care to primary care can be problematic; a key issue is the current shortage of specialists in psychological and behavioural therapies with the necessary training and availability to take referrals from primary care practitioners [8]. Without an adequate broadening of the workforce, GPs have seen a widening in the scope of their role [9]. Not only have primary care physicians increasingly needed to develop psychological intervention skills to manage LTCs and mental health problems, but nurse practitioners are also increasingly becoming involved in delivering psychological interventions to support these patients [10,11].

Primary care nurse-led clinics have proven effective in a providing care for a range of LTCs, including chronic pain [12], medically unexplained symptoms [13], irritable bowel syndrome [14], and diabetes [15]. This is likely to be an increasingly common model for care. Consequently, such therapeutic interventions will be more frequently delivered by generalist primary care nurses with limited training in psychology or mental health.

Both GPs and nurses are generally positive about the increased role of nurse practitioners in chronic disease management, yet it remains unclear how best to implement this change [16]. In particular, it remains unknown what issues confront nurses or their patients. The focus of the present study is to explore changing roles related to a particular LTC for which a growing evidence base of psychological interventions exists: chronic fatigue syndrome/myalgic encephalomyelitis (CFS/ME).

CFS/ME is a symptomatically defined illness with a primary symptom of severe fatigue, unrelieved by rest, and unexplained by medical or psychiatric causes. Fatigue must be present for at least six months and associated with substantial functional impairment [17]. Prevalence estimates are between 0.2% and 0.4% in the UK [18], with substantial economic consequences for services, patients, and their families [19]. By definition, this is an LTC with an average duration between three and nine years and only a minority of patients achieving premorbid levels of functioning [20]. A substantial evidence base now exists as to the most effective ways of managing the condition, with cognitive behavioural therapy (CBT) and graded exercise therapy (GET) having the most robust evidence base [21,22]. In addition, there is evidence that primary care-based counselling involving supportive listening can be as effective as CBT for chronic fatigue [23], though not necessarily for CFS/ME. A counselling approach is relatively more available within primary care [24].

The National Institute for Health and Clinical Excellence (NICE) recommends that like other LTCs, CFS/ME should be managed in primary care, with referral to specialist services only when necessary [25], and preferably locally [18]. However, as for other LTCs, the current shortfall in trained therapists, both within secondary care and primary care, to provide these evidence-based psychological interventions means that NICE recommendations remain unachieved and most CFS/ME patients do not have access to such treatment or must wait, in some cases for several years [20]. Consequently, attention has turned to the possibilities of delivering these interventions through existing non-specialist primary care health professionals [26]. Specifically, it has been identified that PNs are well placed to provide this role [13]. A recent study revealed that nurses themselves would welcome this role, although only if adequately trained and supported [27]. Translating effective treatments from rigorous trial settings to clinical practice can result in more modest effects [28], and similar decrements in effects are found when moving from secondary care to primary care [7]. However, an important first step in maximising the effectiveness embedding a treatment such as nurse-delivered psychological intervention for LTCs into routine clinical practice is to systematically identify potential barriers and solutions necessary to support its introduction [29].

A recent primary care trial that we conducted comparing two nurse-delivered treatments for CFS/ME (FINE trial) [30] provided an opportunity to examine implementation challenges for nurses and patients and to identify strategies to overcome these challenges. Thus, the aim of this study was to identify potential barriers and solutions that could arise if this approach was implemented within routine practice.

## Methods

Our study sample was drawn from patients participating in a randomised controlled trial of two nurse-delivered psychological interventions for CFS/ME in primary care (FINE trial) [30]. Patients (n = 296) for the trial, having been diagnosed with CFS/ME, were referred by their GP and were randomised to receive nurse-delivered supportive listening (SL), pragmatic rehabilitation (PR), or treatment as usual. The focus of the trial was to evaluate the effectiveness of each intervention compared with GP treatment as usual [3]. Interviews for this qualitative study were conducted with the three trial nurses, three supervisors, and a sample of patients. This qualitative study was reviewed and approved by the Eastern Multicentre Research Ethics Committee (MREC) (reference 03/5/62).

Three experienced primary care nurses without specialist training in psychological therapies, and with no prior experience with CFS/ME, were trained to deliver two different evidence-based psychological interventions: PR and SL. PR involves presenting patients with an explanation of their symptoms based on physiological deregulation [31] and encompasses principles of CBT and GET. SL provides emotional support and validation of patients' experiences and is based on a nondirective person-centred counselling approach (See Table 1 for content and structure of each therapy). Each intervention was delivered over 18 weeks with five face-to-face home visits interspersed with five telephone sessions. Sessions lasted between 30 and 90 minutes. A schedule was provided to nurses as a guide to the delivery of PR, although it was recognised that for some patients therapy would proceed more quickly or more slowly or topics might be covered in a different order. Because SL is a patient-led therapy, the patient set the agenda at each session and no schedule of content was provided. Each intervention was supported by a patient manual.

**Table 1:** Structure and content of therapies

	Pragmatic rehabilitation	Supportive listening
**Overview of treatment**	Provides a physiological dysregulation model of CFS/ME, supported by a referenced manual, which underpins the rationale for a programme of graded return to activity, designed collaboratively by patient and therapist. The rehabilitation programme encourages patients to regularise their sleep patterns and includes relaxation exercises to address the somatic symptoms of anxiety and the concentration and memory problems that many patients experience.	A form of nondirective counselling, based on person-centred counselling techniques. The therapist aims to provide an empathic and validating environment in which the patient can discuss his or her concerns and work towards resolution of whichever problems the patient wishes to prioritise.

**Structure of treatment**	Session 1: 90-minute home visit. One-hour home visits on weeks 2, 4, 10, and 18. Thirty-minute telephone calls on weeks 3, 6, 8, 12, and 15.	Session 1: 90-minute home visit. One-hour home visits on weeks 2, 4, 10, and 18. Thirty-minute telephone calls on weeks 3, 6, 8, 12, and 15.

**Content of treatment**	Session 1: Patients are presented with a detailed explanation of their symptoms, supported by a referenced manual, with diary pages, reinforcing the model and outlining a rehabilitative programme. Session 2: The manual is reviewed, patient priorities are determined, and goals for rehabilitation are set collaboratively by the patient and therapist. Care is taken to set goals at a level easily manageable by the patient. Sessions 3-10: At subsequent sessions, progress is reviewed, and the rehabilitative programme adjusted if necessary. Sessions 5-10: Relapse prevention is discussed in the fifth to tenth sessions. In all sessions, the model of CFS/ME contained in the manual is reinforced.	Session 1: The basis of the therapeutic approach is explained and a short booklet with diary pages is given to patients. Issues for discussion in subsequent sessions are elicited, and the therapists use standard counselling techniques of active listening, reflection, and summarizing to ensure that patients feel understood. Sessions 2-10: The therapist summarises the previous session's work and invites the patient to set the agenda for that session's discussion. The therapists do not provide any explanation for patients' symptoms. Throughout, the content of sessions is determined by patients; therapists avoid giving advice or leading patients and concentrate on providing an empathic, validating environment in which patients can discuss their concerns, are encouraged to explore their difficulties, take responsibility for decisions, and identify and use their own resources to manage their health.

CFS/ME = chronic fatigue syndrome/myalgic encephalomyelitis.

All nurses were experienced registered adult-speciality primary care nurses who had no specialist training in mental health, psychology, or CFS/ME. The nurses were female. Nurses were trained to deliver both interventions, in parallel, over a six-month period. Training in each therapy was provided by therapists highly experienced in the particular therapy. Each therapy was also supported by a training manual. For each therapy, nurses received 16 half-days of training, which involved introduction to the techniques and concepts of the therapeutic approach, skills training using role-play and discussions, 'shadowing' trainers in hospital CFS service, and practice with volunteer patients. Sessions with patients were audiotaped (with patients' consent), and the tapes, together with material generated by the patients and the nurses, were assessed to evaluate nurses' practice in accordance with predefined criteria relating to knowledge, skills, and attitude [32]. Each nurse was interviewed individually on two occasions: following training and again following 2.5 years of treatment delivery. Interviews on the first occasion centred on their background, experience of the training, and expectations of delivering therapy. The second interviews focused on experiences of treating people with CFS/ME, delivering the two therapies, and experiences with supervision.

Throughout the trial, the nurses received regular supervision from experienced clinicians with expertise in either PR (n = 2) or SL (n = 1) who had been involved in developing the interventions and protocols and training the nurses. Their professional backgrounds were psychiatry, clinical psychology, and counselling. All supervisors were male. Supervisors met with the therapists frequently (approximately every two weeks for each therapy type), individually or in groups, to discuss and provide advice on individual cases and to ensure therapy was adhering to the relevant protocol. For this study, interviews were conducted with each of the three supervisors towards the end of the trial.

Patients for the trial were recruited from 44 primary care trusts in the northwest of England. Primary care practices were contacted and GPs were invited to refer registered patients with CFS/ME to the trial. Patients were eligible if they were aged 18 years or over, fulfilled the Oxford inclusion criteria for CFS [33], scored 70% or less on the 36 item Short Form Health Survey (SF-36) physical functioning scale, and scored 4 or more on the 11-item Chalder fatigue scale [34]. Following consent, eligible patients were randomised to one of three arms: treatment as usual, SL, or PR. Further details of the trial recruitment procedures are provided elsewhere [30].

Sampling for patients for this qualitative study was purposive and sought to achieve maximum variation in relation to the following: age, gender, deprivation indices, length of time since diagnosis, treatment condition (PR, SL), physical functioning post treatment [35], and level of engagement with therapist [36]. In addition, seven patients who were referred to the trial but declined to participate and six patients who withdrew from the intervention prior to completion were interviewed. Of those who withdrew from the trial, two had not attended any sessions and two attended one session; the final two had attended three and five sessions, respectively. In total, 47 patients were invited to be interviewed; 46 (98%) agreed to take part. Patient interviews were conducted in participants' homes. The final patient sample comprised 33 women (72%); mean age was 46.11 years (range 20-73). Length of time since diagnosis ranged from 1 month to 23 years (mean = 71/2 years).

For each set of interviews, a topic guide provided a flexible framework for questioning and explored a number of areas including: understanding of CFS and its management and nurse-patient relationship and encounters. Patient interviews also explored views of the intervention, whilst supervisor and nurse interviews also explored experiences of delivering the intervention, issues that arose during supervision, and the role and function of supervision. The interviewers combined open questions to elicit free responses, with focused questions for probing and prompting. All interviews were digitally audiotaped and transcribed verbatim.

Nurse interviews lasted between 138 and 180 minutes (mean duration = 159 minutes). Supervisor interviews lasted between 53 and 90 minutes (mean duration = 63 minutes), and participant interviews lasted between 30 and 90 minutes (mean duration = 47 minutes). Interviews with non-participating and withdrawn patients lasted between 20 and 61 minutes (mean duration = 42 minutes).

Analysis proceeded in parallel with the interviews and was inductive (*i.e*., data driven and theory informing, rather than deductive and theory driven), taking an interpretative stance, whereby we sought to explore participants' understanding of their experience through their reports of events [37]. Transcripts were read and discussed by researchers from different professional backgrounds (primary care [n = 2]; psychology [n = 3]; and psychiatry, sociology, and nursing [n = 1 each]), increasing the trustworthiness of the analysis [38]. Coding was iterative and was informed by the accumulating data and continuing thematic analysis. Thematic categories were identified in initial interviews that were then tested or explored in subsequent interviews where disconfirmatory evidence was sought [39]. This was not possible with the other sample categories due to the small number of nurse-therapists and supervisors (n = 3 each). Interviewing the nurses on a second occasion enabled exploration of emerging themes for patients and from initial nurse interviews. Themes were only included in the final analysis if they were common to both therapies, and hence, had the potential to generalise beyond the specific treatment approach. Interpretation and coding of data were undertaken by four of the authors individually, and the themes were agreed on through discussion. Further development of the analysis involved all authors through consensus meetings, whereby interim analysis was presented (along with supporting data) for interrogation by the wider team. Ideas emerging from these discussions could then be subsequently tested within further interviews and analysis.

In reporting the final analysis, the data are presented to illustrate the range and commonality of meaning of each category of analysis from the perspectives of patients, nurse therapists, and supervisors.

## Results

**Figure 1:**
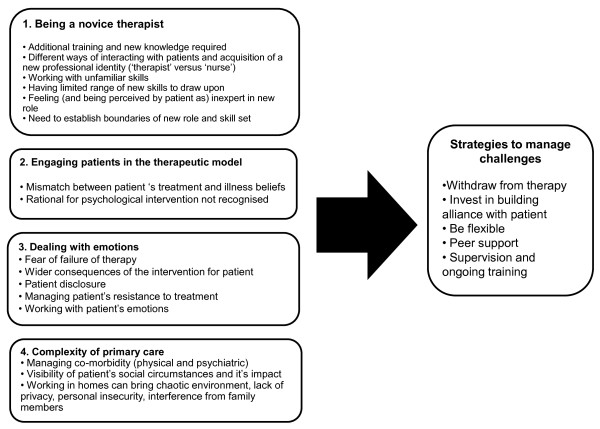
**Challenges of nurse delivery of psychological interventions and strategies developed by nurses and patients**.

Four sets of challenges emerged that led to, or had potential to lead to, tension between patients and therapists and were common to both interventions. These are considered in turn: (i) being a novice therapist, (ii) engaging patients in the therapeutic model, (iii) dealing with emotions, and, (iv) the complexity of primary care. The analysis and interpretation of the data are extended to demonstrate the strategies nurses and patients employed to manage these tensions (see Figure 1 for summary of analysis).

### (i) Being a novice therapist

Whilst the nurse therapists were highly experienced as nurses (having each been registered for over 20 years as a nurse and worked at least 15 years in primary care), the role of therapist was novel. Training for the two therapy interventions was substantial (approximately six months full-time) and was found to be demanding, yet this was contrasted with their extensive experience in their nursing role.

*'Although they had the training of course but it didn't compare to the lifetime's experience they have had of working in nursing.' *(Supervisor)

Working as a therapist and delivering therapy was described as very different to their experiences of delivering nursing care. Nurses were aware they had to behave differently, and this took additional effort on their part.

*'*[As] *a nurse...I would have just turned up, said "right get on the bike. I will talk you through it" and that is something that I have had to restrain myself with...I really had to teach myself to step back.' *(Nurse)

Supervisors saw that, particularly during the first year of delivering therapy, they were required to help nurses develop into their new identity, relating to patients in a different way.

*'They felt they were nurses and they felt, not frauds exactly working as counsellors, but you know not at home in that field really. I think part of my role was a kind of normative role helping them to feel their way into the persona really of being a counsellor...rather than just dealing with the nitty gritty of the patients.' *(Supervisor)

Despite their newly found knowledge and skills in these two specific therapies, not having a background as psychological or behavioural therapists meant that the nurses had a relatively limited range of therapeutic skills to draw upon, particularly for more challenging or complex cases.

*'They don't know the kind of therapeutic tricks which you have, which you pick up from being a therapist.' *(Supervisor)

Inevitably, there were aspects of their new role they felt more comfortable and familiar with.

*'We selected them for being general nurses, not mental-health trained...they are less comfortable with this *[patients' anxieties] *than with the physical reconditioning or even mental reconditioning.' *(Supervisor)

The role of novice therapist was frequently contrasted with the role of the expert patient. Many patient participants had had CFS/ME for many years and were highly informed about the illness and management options.

*'With having ME for nearly 10 years I have read a lot about it and you know I had talked to the doctor about it.' *(Patient, Supportive Listening group)

Patients were also very familiar with their symptoms and had identified patterns of triggers and ways of managing their condition. This expertise enabled them to feel able to disagree with and challenge the nurse therapist.

*' "I don't want to fall out with you," she said... There was nought wrong with her *[therapist], *we just didn't agree on what she said.' *(Patient, Supportive Listening group, withdrew from therapy)

'*She *[nurse-therapist] *was on about sleep. I have to get up early in the morning and I said, "Well when you have only had a couple of hours sleep, you can't can you," we were arguing about that... I will go to bed, it will take me about three hours to get to sleep, and I will have an hour, and I will waken up, and it will take me another three hours, to get off again. "Well you should get up like at 9 o'clock every morning, and then you will be more tired, you know, and you would sleep better when you went to bed". I said, "No it doesn't work like that", I said "I have tried that before". So we were falling out about that.' *(Patient, Pragmatic Rehabilitation group, withdrew from therapy)

Having to work with patients who often were already highly informed about their LTC was challenging when having to work in a new way, and nurses felt they were under a high degree of scrutiny by patients.

*'They are testing you all the time.' *(Nurse)

This led nurses to feel their expertise as therapists was not automatically established and, furthermore, was sometimes brought into question.

*'She happened to be on the SL arm, you know, of the trial and very sort of insulting to me really*.. [she said to me] *"Are you actually trained?" *[I thought] *"No I have just come off Tesco's cash till!" And really, really she would try and goad me... "Are you sure you have been trained?" and "What exactly have you read up on?" and, you know, things like this.' *(Nurse)

Consequently, nurses had to learn the limitations and boundaries of their new role, and supervision was vital to support them in this.

*'I think one of the skills that they have developed and that we have talked about is the skill of knowing when not to open something up with a patient, because you know you haven't got the resources, the scope to deal with it.' *(Supervisor)

### (ii) Engaging patients in the therapeutic model

A critical component of successfully engaging a patient in a treatment is to ensure they understand and accept the rationale for the treatment [40] Understanding and acceptance were not synonymous in our sample, as the following two quotations demonstrate:

*'She explained all about...the physiology of it...first time that I understood why my energy was so low; made a lot of sense.' *(Patient)

*'It *[the PR intervention] *insisted that physiologically there was nothing wrong. There was nothing wrong with my glands, there was nothing wrong, that it was just deconditioned muscles. And I didn't believe that...I can't get well with treatment you don't believe in.' *(Patient, declined to participate in the trial)

As with all long-term physical conditions, some individuals do not necessarily recognise the rationale for psychological intervention. This is particularly true for CFS/ME, where there are a wide range of illness beliefs about the etiology and nature of CFS/ME [41]. Consequently, it was expected by the nurses that the therapy would pose a challenge for some patients and had potential to create some conflict and this indeed was reported by the nurses.

*'You might have a little bit of a tussle for the first couple of weeks while they are getting their head around the concept.' *(Nurse)

However, an unresolved mismatch between patient's illness and treatment beliefs was a key source of tension. Evidence for this was found for both PR and SL treatments.

*'If all that was standing between me and recovery was the reconditioning I could work it out and do it, but what I have got is not just a reconditioning problem. I have got something where there is damage and a complete lack of strength actually getting into the muscles and you can't work with what you haven't got in terms of energy*.' (Patient, Pragmatic Rehabilitation group)

*'I mostly believe it was more physical than anything else, and I didn't see how talking could truthfully, you know, if it was physical, do anything.' *(Patient Supportive Listening group)

At times, this lack of agreement over the nature of the condition and lack of acceptance as to the rationale behind the treatment led to conflict.

*'I kept arguing with her all the time because I didn't agree with what she said.' *(Patient Pragmatic Rehabilitation group, withdrew from therapy)

Conversely, when patients who had expressed initial resistance were effectively engaged with the programme and therapy could progress, this was enormously rewarding for the nurses.

*'And it was like watering a flower it was really lovely for me personally... it was lovely watching her just blossom, you know what I mean, because she finally took on board the physical stuff and the sleep.' *(Nurse)

### (iii) Dealing with emotion

A further set of challenges arose from the emotional aspects of the therapy. Firstly, nurses had a range of their own concerns and emotions that they had to manage, which included learning to manage potential (and occasionally actual) failure.

*'Therapist said to me, "Is this working for you?" and I said, "No it's not". She took that very personally. I wasn't aggressive at all.' *(Patient, Supportive Listening group)

*'One common theme I think which has come up is the difficulty of accepting that you can't get it right all the time.' *(Supervisor)

Secondly, whilst their focus was on treating CFS/ME with a view to improving the symptoms of the condition, nurses were aware that there were wider consequences of their intervention, which could be a cause for worry.

*'I just hope she doesn't get a divorce...I am frightened in case I open up a can of worms...I don't want to leave an aftermath.' *(Nurse)

This was of particular concern since the therapy, as is common within a primary care service, was for a relatively short prescribed period of time.

*'We had patients where somebody will disclose to the nurse therapist in the first session that they were sexually abused as a child...in conventional therapy that is unusual, you would normally expect somebody not to reveal something like that until they have really established a good, trusting relationship. But sometimes people are so desperate, you know, they have been holding this secret for 20, 30 years and here is somebody offering the chance of a trusting relationship; I will risk it. So then the material is out, you can't put it back...They *[nurses] *have talked about issues like that and how to, how to deal with something as deep as that, when you have only got very few sessions.' *(Supervisor)

*'This amount of counselling is dangerous. Because what it does do is it opens up and it's not long enough to deal with the scratch, so what you are doing is you are scratching the top off. I mean, not because I am going to fall apart at the seams, if it was for someone else, then by the time you got to this point in the time and it's finishing you have only just scratched the surface but you started the process.' *(Patient, Supportive Listening group)

A particularly difficult challenge of interacting with patients for the nurses and their supervisors was managing patients' resistance to the treatment. This arose from patients not accepting the rationale for the treatment and occurred for both types of psychological treatments, though for different reasons.

*'I used to go there and she would totally block me, she would sit with her arms folded, total silence in the house...she pulled out of the trial...it was tortuous for both of us.' *(Nurse)

*'There have been one or two times where I have been worried because they have got angry at the patients...that anger has been communicated to the patients. Their frustration has reached the point where they sort of boiled over... there is sort of feeling that the patient should be grateful and follow your advice, and in actual fact, what happens is the patient is quite resistant and there is this thing like you know, "The bastards don't want to get better"...I think it's a difficult thing for all therapists and I think basically over the time you just basically learn to cope with it, and but they have not had time.' *(Supervisor)

Managing patients' emotions was an extremely demanding aspect of their new role.

*'That anger...it's very wearing and demoralizing.' *(Nurse)

### (iv) Complexity of primary care

A further set of challenges arose from working within the context of primary care. Within the sample there was considerable comorbidity, including both physical and mental health problems as ascertained with the help of their supervisors. Medical health problems appeared common and required nurses to tailor therapy (*e.g*., exercise advice) or advise patients to seek additional medical treatment; this was described by nurses as fitting comfortably within their expertise, and they were able to draw upon their experience. Having a background in primary care medicine meant they were comfortable knowing when and where to obtain help for medical-related problems and making judgements about risk. More challenging were patients' mental health problems, which felt less familiar and on occasion daunting to the nurses. Patients often also had social circumstances that impacted therapy (*e.g*., housing or relationship difficulties). This is not to say that a primary care sample necessarily has a greater level of social problems, rather, that by working with patients within this setting, these problems were more visible to the nurse.

*'There are a lot of differences seeing things from the primary care angle...you have to deal with more uncertainty...you also really do hear accounts which are somewhat sanitised when you see them in outpatients...you get a much more direct assessment of their life and this affects the whole flavour of what one does, you put more weight to the social circumstances if you are more directly aware of them. Seeing them in outpatients you become much more detached and then offer them a rigid programme.' *(Supervisor)

Nurses were even more aware of how patients were functioning and the circumstances they were dealing with since therapy was delivered in patients' own homes. This was thought to be helpful in building the therapeutic relationship.

*'Going into their homes, you are in their territory, and they are more comfortable coping with the intervention because it is adaptable to the person's needs, and I think that has probably helped reduce conflict for the whole study.' *(Supervisor)

However, it also meant the nurse therapists needed to create additional boundaries over how they worked and had to manage distractions, which could include chaotic or deprived environments, lack of privacy, interference from family members, and issues of personal security.

*'Whole load of practical issues about homes, which are sometimes disruptive, there are partners creeping around, sometimes, they keep interfering with what is going on. The nurses find that uncomfortable sometimes, going into homes... sometimes they are very squalid...which is not conducive to being a good therapist.' *(Supervisor)

*'When they *[family members] *are hovering around in the background, I would rather have them in the room with me, hearing what is going on, than walking up and down and listening outside. Particularly when something has been said about them which is really uncomfortable...where they are blaming, you know, the other person for half of the condition or, you know, blaming the trigger factors on other family members and things like that.' *(Nurse)

### Strategies for managing tensions

A number of strategies were developed and identified by all parties to avoid or manage these potential tensions (see Figure 1). Patients could (and occasionally did) drop out of treatment: 19 out of 183 (9.6%) participants withdrew after starting treatment [42]. Unless there were clinical reasons why it was appropriate, nurses were unable to discontinue treating a patient simply due to tensions. However, they did describe cases where they had to just 'get through'.

*'Thinking about individuals where this type of therapy isn't helping, this is cases that we have had through the trial...sometimes it's somebody that you just can't, you get annoyed with, and you just think, right, let's just ride it out, we are not going to change things.' *(Nurse)

Nurses recognised that it was important to invest in building a therapeutic alliance to engage patients, in particular, explaining the rationale for the treatment and listening to and validating patients' illness experience. This was highly valued by patients.

*'What I found useful was not necessarily her specific knowledge in terms of chronic fatigue syndrome, but her ability to spend time listening to me and helping me with my issues. I think first and foremost who she was, her empathic nature, was her greatest skill, anything else for me came secondarily,' *(Patient, Supportive Listening group)

Another important strategy for engaging patients was to build in flexibility by, for example, re-ordering the programme for individuals.

*'Very early on we realised that...do things that they are going to get quick gain from and get them on board, rather than giving them a challenge that they are so frightened of that they won't do any of the programme.' *(Nurse)

Nurses also reflected that flexibility was limited within the trial setting (where treatment protocols were more tightly constrained) and that within a clinical setting there would be options to defer treatment until a more suitable time for the patient or to do some preparatory work to help the engage the patient, such as providing more sessions for more complex cases or choosing one treatment over another. Patients also found ways to build flexibility into their treatment.

*'I have changed it and I have devised my own, I am doing it in my own way.' *(Patient Pragmatic Rehabilitation group)

Supervision was viewed by nurses and supervisors as fundamental in managing tensions arising from delivering the therapy. Within supervision, nurses were helped to formulate tensions that had arisen in order to understand what might be the causes and create potential solutions for overcoming these.

*'I get them to think about why they have this feeling about a patient and how not to let it interfere with the start of the treatment and move it on... get them to look at how they feel, use how they feel to work through the problem, see where the sticking point is for them and see if that helps, as you say, unpick it; is it a problem with the person-to-person interaction or is it resistance?' *(Supervisor)

In some circumstances, this tension could be put to use and provided opportunities for therapists to understand further a patient's experience and engage them.

*'You get quite an interesting phenomenon because you get the patient being angry with the therapist, because the therapist isn't giving them the therapy they hoped they would get. That actually can be quite a useful vehicle for exploring what is going on in the patient's mind, what goes on in relationships in their family, what part anger plays in their life really, erm, so it's not necessarily a bad thing, but it does happen quite a lot.' *(Supervisor)

The type of supervision experienced was highly valued and was viewed as qualitatively different from the supervision experiences they received whilst working in non-mental health settings.

*'I am well aware of different models of supervision, and aware that, say, a CPN *[community psychiatric nurse] *will have management supervision as well as clinical supervision and they *[general adult nurses] *are more used to having management supervision, which is quite prescriptive and quite directive and persecutory quite often...I certainly think they have found this very, I believe they have found it very supportive...I think they found it pleasantly different.' *(Supervisor)

The nurses also made use of having peers with the same skills and experiences. From this they derived emotional support and generated new ideas to manage challenging cases.

*'If we were having a really difficult time with a certain patient, then we would sort of pool ideas, and ask advice how they would cope with it, the other nurse therapists or what do they think is going on.' *(Nurse)

Of all the strategies identified, effective supervision was seen by the nurses as overwhelmingly the most useful way of resolving the challenges identified.

*'All of them were brilliant, they really were, I couldn't have done without it really. I would have left probably...I probably would have got another job actually without the supervision, because it was too hard, too hard at times.' *(Nurse)

## Discussion

This study identified implementation issues for nurse-led psychological interventions for LTCs., This paper examined data from interviews conducted with therapists, supervisors, and patients, during a randomised controlled intervention study comparing two evidence-based psychological treatments for CFS/ME delivered by non-specialist nurses. Both interventions comprised therapeutic approaches known to be effective for other LTCs, currently available in primary care, and that have potential to have an increasing role in primary care nursing. To our knowledge this is the first study to systematically examine implementation concerns raised in relation to non-specialist nurse delivery of treatment for a complex and challenging LTC--CFS/ME. We achieved this by selecting experienced nurses, new to delivering these therapies, working in a well-supported clinical trial context. Several challenges emerged common to both therapies that had the potential for tension and even conflict if implemented into routine practice. These challenges arose from the practitioners' changing role, the demanding nature of delivering therapy and working with clients, and the organisational context within which it was being delivered.

Working as a therapist is a qualitatively different role to being a nurse, requiring adjustments to a new identity, behaviours, and ways of interacting with patients. At times, this created concern for the nurses that their expertise was questioned by patients who, in contrast, had an unquestionable authority due to their long-term experience of their symptoms [43] and knowledge of the condition. According to independent ratings in a related study, the quality of the therapy delivery was satisfactory to good [42]. However, our data suggest that experienced general nurses lack the breadth of training in psychological treatments that would help with more challenging cases. If the therapy had been conducted by a competent psychotherapist without a professional background in general medicine or primary care, different challenges would likely have arisen. Having a nursing background conferred clear advantages. It is likely that no single group of workers are ideal for the role of providing psychological treatment for patients with LTCs. For effective implementation it will be important to promote realistic expectations regarding the level and range of skills in delivering complex psychological and behavioural interventions that can be learnt in relatively short periods.

Managing patients' illness beliefs and resistance to psychotherapy was a consistent theme across nurse, supervisor, and patient interviews. A similar finding has emerged from non-specialist nurse delivery of CBT for high-utilising patients with medically unexplained symptoms [13]. In both studies, nurses found patient beliefs and resistance to be the most emotionally challenging aspect of delivering psychological therapies. This is not surprising since dealing with resistance is a theme that is common in supervisions [44]. For some patients, these challenges were mild enough so that they could be negotiated or even used to some advantage. Nurse satisfaction with their role as therapists was derived when such instances were successfully negotiated. Through expert and peer supervision, nurses learnt that investing time in building a trusting therapeutic relationship was critical. Ensuring illness beliefs are aligned with the therapeutic approach was essential to engaging patients in treatment, a finding that is not peculiar to medically unexplained conditions, but is common across a wide range of physical and mental health problems [40,45]. However, our previous work with treating CFS/ME demonstrated that when therapists and referrers are able to successfully align treatment and illness beliefs, patients are able to successfully engage in and complete therapy [46].

The environment within which therapy took place in itself provided a challenge and had the potential to cause tension. Patients with CFS/ME were referred to the treatment trial directly by their GP rather than, as might be typical in a secondary care setting, after screening by a specialist. This resulted in a heterogeneous sample with a wide range in levels of disability and mental health and medical comorbidity; over half of the trial sample reported one or more medical conditions that did not explain their fatigue, with the most common being musculoskeletal, gastrointestinal, or cardiovascular conditions [42]. Despite high levels of additional medical problems, nurses proved able to tailor therapy to account for these problems. This is an advantage of employing non-specialist nurses who have expertise working with medical problems, and this was an area the nurse therapists felt confident and familiar with. In contrast, managing psychiatric comorbidity was more challenging, and nurses frequently sought supervisors' expertise on mental health matters. Indeed, the most appropriate role for the non-specialist nurses may be to work with cases where medical problems predominate and to identify more complex cases where specialist mental health intervention is required and facilitate an appropriate stepped referral [11].

Working in primary care, particularly within a domiciliary service, increased the proximity of the therapist to the social context within patient lived. Elsewhere, this has been shown to challenge nurses with having to choose either a 'guest' or 'professional' position during interactions in patients' homes [47]. At times social circumstances hindered therapy considerably. In another study we found the number of social problems and level of social support to be associated with adherence to the therapy [48]. These findings might help explain the reduction in consistency and effect sizes for primary care treatments compared to those in secondary care [7]. The complexity of working in primary care led nurses to apply therapy more flexibly than might be the case within a secondary care service, a strategy that should be considered when translating secondary care interventions to primary.

This study systematically gathered data from three different perspectives. This is an effective way of increasing the trustworthiness of the analysis [38]. A further strength of the study was the high level of recruitment, with 98% of patients approached agreeing to take part in the qualitative interviews. Purposive sampling enabled us to access a wide range of views. In particular, and unusually, the sample included participants who had chosen not to take part in, or had withdrawn from, therapy. This allowed us to examine the views of those where tensions and conflict may have been greatest, resulting in breakdown of the relationship (*e.g*., patients who had withdrawn from treatment). Patient attrition is often higher when implementing psychological interventions outside the resources of a research trial [49] so these are critical data to inform future developments.

A limitation of the study was the small sample of nurse therapists and supervisors available for interview, although we interviewed all those involved in the trial and they all had remained in post throughout the study. These interviews were lengthy and rich (and in the case of the nurses, were repeated on two occasions to capture evolution in their views), and themes were only included in the final analysis where corroboratory evidence was found from the very substantial patient data set.

All the patients in this study had CFS/ME, and it is possible that the challenges found may not generalise to patients with other LTCs. However, as a condition, CFS/ME combines a number of features that are common to other LTCs, including a range of physical symptoms that impact on a number of domains and functioning, wide range of illness beliefs, and comorbidity with other physical and mental health conditions. Furthermore, challenges were only included in the analysis and reported here when they had potential to be relevant to non-CFS/ME conditions. Nevertheless, whether the same challenges face nurses delivering other therapies to other patient groups is an empirical question and further research is needed for clarification.

An additional potential limitation is that we only studied the experiences of delivering two psychological interventions, and additional or different challenges may emerge for other treatment approaches. PR (combining aspects of CBT and GET) and SL represent the treatment approaches with the strongest evidence base for this LTCs, treatment approaches that are recommended by NICE (25) and so are most likely to be utilised in future clinical services within routine primary care. However, the effects of PR within the trial compared to usual GP care were modest, and in the case of SL, not significantly beneficial [42]. It is possible that the challenges identified within this study were peculiar to these particular treatment approaches and fewer challenges may have arisen within a trial that had demonstrated greater effects. However, since evidence for all challenges were found across treatments and we purposively sought to include participants in each arm who had a good outcome as well as those who had not benefited from each intervention, this is unlikely. Indeed, since effect sizes are generally diluted when implementing treatments to routine practice [49], it is probable that the tensions we identified would be more pronounced outside a trial setting.

The amount and type of supervision the nurses received was not typical of what would be experienced in routine primary care. Our therapists received clinical supervision every two weeks by experts in each treatment approach. This supervision 'best practice' has been recognised as a key reason why effects from research findings often fail to translate to clinical practice [49]. Primary care nurses within routine practice recognise that supervision is a core prerequisite for effective delivery of psychological interventions in primary care but are sceptical that training and ongoing support would be sufficient [27]. Where available (here in a research trial) the nurses made great use of, and highly valued, their supervisors' expertise to help them address and resolve tensions. Outside of research settings, clinical supervision is a valued and effective method for developing nurses' knowledge, skill, professional accountability, and for reducing staff burnout [50]. However, this type of supervision is far more common in the mental health and aged care compared with physical health or primary care nursing [50]. Hence it is unclear how likely it is that general nurses working within a primary care setting would have regular access to high-quality clinical supervision. Successful implementation of nurse-delivered psychological interventions for complex problems has utilised high levels of expert psychological supervision [13]. The costs and time needed to train nurses is significant, and effective supervision can play a role in retention of therapists, an issue that has hindered previous implementation efforts [49]. Future research should examine the effectiveness and cost effectiveness of adequately supported nurse-led psychological interventions and identify ways of delivering this across primary care settings.

The creation and resourcing of appropriate supervision is a fundamental issue to be addressed if nurses are expected to effectively deliver psychological interventions to patients with complex LTCs within primary care. This role may prove a more efficient use of the limited resources within primary care. There is also the potential for peer supervision to support this process [51], though this would require experienced nurses to be working in this way within a given locality.

## Conclusions

Expanding the role of non-specialist primary care nurses to include delivering psychological interventions for patients with LTCs such as CFS/ME creates a number of challenges. Quality clinical supervision to support nurses is necessary if this is to become a feasible practice.

## Competing interests

The authors declare that they have no competing interests.

## Authors' contributions

SP and CCG designed and managed the qualitative study. They contributed to the data collection, analysis, and early drafts of the paper. SP is guarantor for the study and paper. AW was principle investigator for the trial from which participants were recruited. GC and JB collected data and contributed to the analysis. AW, RM, KL, and CFD contributed to data analysis and revising the paper. All authors read and approved the final manuscript.

## References

[B1] Roland M Sibbald B McDonald R Care closer to home. Moving care Health Service Journal 2007 117 6065 6 8 17844859

[B2] Department of Health The NHS Plan 2000 London: Department of Health

[B3] Coventry P Gellatley J Improving outcomes for COPD people with mild-to-moderate anxiety and depression: a systematic review of cognitive behavioural therapy British Journal of Health Psychology 2008 13 381 400 17535503 10.1348/135910707X203723

[B4] Dixon KE Keefe FJ Scipio CD Perri LM Abernethy AP Psychological interventions for arthritis pain management in adults: A meta-analysis Health Psychology 2007 26 3 241 250 17500610 10.1037/0278-6133.26.3.241

[B5] Morley SJ Eccleston C de C Williams AC Systematic review and meta-analysis of randomised controlled trials of cognitive behaviour therapy and behaviour therapy for chronic pain in adults, excluding headache Pain 1999 80 1 13 10.1016/S0304-3959(98)00255-3 10204712

[B6] Hoffman BM Papas RK Chatkoff DK Kerns RD Meta-analysis of psychological interventions for chronic low back pain Health Psychology 2007 26 1 9 17209691 10.1037/0278-6133.26.1.1

[B7] Raine R Haines A Senskey T Hutchings A Larkin K Black N Systematic review of mental health interventions for patients with common somatic symptoms: can research evidence from secondary care be extrapolated to primary care? British Medical Journal 2002 325 1 11 10.1136/bmj.325.7354.1 12424170 PMC131187

[B8] Trude S Stoddard JJ Primary care physicians and mental health services Journal of General Internal Medicine 2003 18 442 449 10.1046/j.1525-1497.2003.30216.x 12823651 PMC1494870

[B9] St Peter RF Reed MC Kemper P Blumenthal D Changes in the scope of care provided by primary care physicians The New England Journal of Medicine 1999 26 1980 1985 10.1056/NEJM199912233412606 10607816

[B10] Gemmell I Campbell S Hann M Sibbald B Assessing workload in general practice in England before and after the introduction of the pay-for-performance contract Journal of Advanced Nursing 2009 65 3 509 515 10.1111/j.1365-2648.2008.04902.x 19222648

[B11] Katon W Von Korff M Lin E Simon G Rethinking practitioner roles in chronic illness: the specialist primary care physician, and the practice nurse General Hospital Psychiatry 2001 23 138 144 10.1016/S0163-8343(01)00136-0 11427246

[B12] Glasgow ACA Glasgow JA Development of a nurse-led chronic pain clinic in UK primary care International Journal of Clinical Practice 2002 56 1 21 25 11833551

[B13] Lyles JS Hodges A Collins C Lein C Given CW Given B Using nurse practitioners to implement an intervention in primary care for high-utilizing patients with medically unexplained symptoms General Hospital Psychiatry 2003 25 63 73 10.1016/S0163-8343(02)00288-8 12676418

[B14] Kennedy T Jones R Darnley S Seed P Wessely S Chalder T Cognitive behaviour therapy in addition to antispasmodic treatment for irritable bowel syndrome in primary care: randomised controlled trial British Medical Journal 2005 331 7514 435 437 10.1136/bmj.38545.505764.06 16093252 PMC1188111

[B15] Whittlemore R Melkus GD Sullivan A Grey M A nurse-coaching intervention for women with type 2 diabetes Diabetes Educator 2004 30 5 795 804 10.1177/014572170403000515 15510531

[B16] Sciamanna CN Alvarez K Miller J Gary T Bowen M Attitudes toward nurse practitioner-led chronic disease management to improve outpatient quality of care American Journal of Medical Quality 2006 21 6 375 381 10.1177/1062860606293075 17077419

[B17] Fukuda K Straus SE Hickie I Sharpe MC Dobbins JG Komaroff A The international chronic fatigue syndrome study group. The chronic fatigue syndrome: a comprehensive approach to its definition and study Annals of Internal Medicine 1994 121 12 953 959 7978722 10.7326/0003-4819-121-12-199412150-00009

[B18] Department of Health CFS/ME Working Group Report of the Report to the Chief Medical Officer of an Independent Working Group 2002 London

[B19] McCrone P Darbishire L Ridsdale L Seed P The economic cost of chronic fatigue and chronic fatigue syndrome in UK primary care Psychological Medicine 2003 33 2 253 261 10.1017/S0033291702006980 12622304

[B20] Cairns R Hotopf M A systematic review describing the prognosis of chronic fatigue syndrome Occupational Medicine 2005 55 20 31 10.1093/occmed/kqi013 15699087

[B21] Price JR Mitchell E Tidy E Hunot V Cognitive behaviour therapy for chronic fatigue syndrome in adults Cochrane Database of Systematic Reviews 2008 3 10.1002/14651858.CD001027.pub2 PMC7028002 18646067

[B22] Chambers D Bagnall AM Hempel S Forbes C Interventions for the treatment, management and rehabilitation of patients with chronic fatigue syndrome/myalgic encephalomyelitis: an updated systematic review Journal of the Royal Society of Medicine 2006 99 10 506 520 10.1258/jrsm.99.10.506 17021301 PMC1592057

[B23] Ridsdale L Godfrey E Chalder T Seed P King M Wallace T Chronic fatigue in general practice: is counselling as good as cognitive behaviour therapy? A UK randomised trial British Journal of General Practice 2001 51 462 19 24 11271868 PMC1313894

[B24] Mellor-Clark J Simms-Ellis R Burton M National Survey of Counsellors in Primary Care: Evidence for Growing Professionalisation Occasional Paper 79. London: Royal College of General Practitioners 2001 PMC2560969 19790944

[B25] NICE CF 53 Chronic fatigue syndrome/myalgic encephalomyelitis (or encephalopathy): guideline 2007 London

[B26] Huibers M Beurskens A Van Schayk C Bazelmans E Metsemakers J Knottnerus J Bleijenberg G Efficacy of cognitive-behavioural therapy by general practitioners for unexplained fatigue among employees. Randomised controlled trial British Journal of Psychiatry 2005 184 240 246 10.1192/bjp.184.3.240 14990522

[B27] Chew-Graham C Dixon R Shaw JW Smyth N Lovell K Peters S Practice nurses' views of their role in the management of chronic fatigue syndrome/Myalgic Encephalitis: a qualitative study BMC-Nursing 2009 8 2 10.1186/1472-6955-8-2 19161604 PMC2635361

[B28] Rothwell PM Can overall results of clinical trials be applied to all patients? Lancet 1995 345 1616 1619 10.1016/S0140-6736(95)90120-5 7783541

[B29] May C A rational model for assessing and evaluating complex interventions in health care BMC - Health Services Research 2006 6 86 10.1186/1472-6963-6-86 16827928 PMC1534030

[B30] Wearden AJ Riste L Dowrick C Chew-Graham C Bentall R Morriss R Peters S Dunn G Richardson G Powell P Fatigue Intervention by Nurses Evaluation - The FINE Trial. A randomised controlled trial of nurse led self-help treatment for patients in primary care with chronic fatigue syndrome: study protocol [ISRCTN74156610] BioMed Central-Medicine 2006 http://www.biomedcentral.com/1741-7015/4/9 10.1186/1741-7015-4-9 PMC1456982 16603058

[B31] Powell P Bentall RP Nye FJ Edwards RHT Randomised controlled trial of patient education to encourage graded exercise in chronic fatigue syndrome British Medical Journal 2001 322 7283 387 390 10.1136/bmj.322.7283.387 11179154 PMC26565

[B32] Morriss R Gask L Smith C Battersby L Training practice nurses to assess and manage anxiety disorders: A pilot study Nursing Times Research 1999 4 132 142

[B33] Sharpe M Archard L Anatvala J Borysiewicz L Clare A Edwards R Hawton K Lambert H Lane R McDonald E Mowbray J Pearson D Peto T Preedy V Smith A Smith D Taylor D Tyrrell D Wessley S White P A report - Chronic fatigue syndrome - guidelines for research Journal of the Royal Society of Medicine 1991 84 118 121 1999813 10.1177/014107689108400224 PMC1293107

[B34] Chalder T Berelowitz G Pawlikowska T Development of a fatigue scale Journal of Psychosomatic Research 1993 37 2 147 153 10.1016/0022-3999(93)90081-P 8463991

[B35] Ware JE Sherbourne CD The MOS 36-item short-form health survey (sf-36). 1. conceptual-framework and item selection Medical Care 1992 30 6 473 483 10.1097/00005650-199206000-00002 1593914

[B36] Muran JC Gorman BS Safran JD Twining L Samstag LW Winston A Linking in-session change to overall outcome in short-term cognitive therapy Journal of Consulting and Clinical Psychology 1995 63 651 657 7673543 10.1037//0022-006x.63.4.651

[B37] Malterud K Qualitative research: standard, challenges and guidelines Lancet 2001 358 483 488 10.1016/S0140-6736(01)05627-6 11513933

[B38] Peters S Qualitative methods and mental health research Evidence Based Mental Health 2010 13 35 40 10.1136/ebmh.13.2.35 21856603

[B39] Strauss A Corbin J Basics of Qualitative Research: Techniques and Procedures for Developing Grounded Theory 1998 2 Sage, Thousand Oaks, CA

[B40] Moss-Morris R Petrie K Weinman J Functioning in chronic fatigue syndrome: do illness perceptions play a regulatory role? British Journal of Health Psychology 1996 1 15 21 10.1111/j.2044-8287.1996.tb00488.x

[B41] Wearden AJ Dowrick C Chew-Graham C Bentall RP Morriss RK Peters S Riste L Richardson G Lovell K Dunn G Fatigue Intervention by Nurses Evaluation - The FINE Trial. A randomised controlled trial of a nurse-led home-based self-help treatment for patients in primary care with chronic fatigue syndrome [ISRCTN 74156610] British Medical Journal 2010 340 c1777 10.1136/bmj.c1777 16603058 PMC1456982

[B42] Peters S Stanley I Rose M Salmon P Patients with medically unexplained symptoms: sources of patients' authority and implications for demands on medical care Social Science and Medicine 1998 46 4-5 559 565 10.1016/S0277-9536(97)00200-1 9460835

[B43] Marshall AA Smith RC Physicians' emotional reactions to patients: recognizing and managing countertransference American Journal of Gastroenterology 1995 90 4 8 7801947

[B44] Leventhal H Diefenbach M Leventhal EA Illness cognition: using common sense to understand treatment adherence and affect cognition interactions Cognitive Therapy and Research 1992 16 143 163 10.1007/BF01173486

[B45] Hagger MS Orbell S A meta-analytic review of the common-sense model of illness representations Psychology & Health 2003 18 141 18422201156

[B46] Chew-Graham C Brooks J Wearden A Dowrick C Peters S Factors influencing engagement of patients in a novel intervention for CFS/ME: a qualitative study Primary Health Care Research & Development 2010 1 11 21457596 10.1017/S146342361000037X

[B47] Oresland S Maatta S Astrid N Jorgensen MW Lutzen K Nurses as guests or professionals in home health care Nursing Ethics 2008 15 3 371 383 10.1177/0969733007088361 18388171

[B48] Morriss R Bennett C Fleetwood D Connell S Bentall RP Riste L Wearden A Adaptations and adherence to pragmatic rehabilitation delivered to patients with chronic fatigue syndrome by general nurses in primary care. (under submission)

[B49] Scheeres K Wensing M Knoop H Bleijenberg G Implementing cognitive behavioural therapy for chronic fatigue syndrome in a medical health center: a benchmarking evaluation Journal of Consulting and Clinical Psychology 2008 76 1 163 171 18229994 10.1037/0022-006X.76.1.163

[B50] Brunero S Stein-Parbury J The effectiveness of clinical supervision in nursing: an evidenced based literature review Australian Journal of Advanced Nursing 2007 25 3 86 94

[B51] Teasdale K Brocklehurst N Thom N Clinical supervision and support for nurses: an evaluation study Journal of Advanced Nursing 2001 33 2 216 224 10.1046/j.1365-2648.2001.01656.x 11168705

